# Effect of social disparities on 10 year survival in pediatric patients with Wilms' tumor

**DOI:** 10.1002/cam4.5124

**Published:** 2022-08-09

**Authors:** Victor Chalfant, Carlos Riveros, Andrew A. Stec

**Affiliations:** ^1^ Department of Urology Creighton University School of Medicine Omaha Nebraska USA; ^2^ Department of Urology University of Florida Health Jacksonville Florida USA; ^3^ Division of Urology Nemours Children's Health Jacksonville Florida USA

**Keywords:** social deprivation index, social determinants of health, socioeconomic status, survival outcomes, Wilms' tumor

## Abstract

**Background:**

To stratify 10‐year survival outcomes by degree of social disparities in pediatric Wilms' tumor patients. We applied the Social Deprivation Index (SDI) to survival outcomes from the national SEER database to elucidate the effects of lower socioeconomics on cancer survival.

**Methods:**

A retrospective cohort study was performed using the national Surveillance, Epidemiology, and End Results (SEER) oncology registry from 1975 to 2016 based on county‐level data. Pediatric patients (<18 years old) with a diagnosis of WT (C64.9) and confirmed based on histology codes (8960/8963) were included. SDI scores were calculated for each patient and initially divided into quintiles. Patients were delineated into high‐risk (>60th percentile/more deprived) or low‐risk (<60th percentile/less deprived) groups. Statistics were assessed using Fisher's exact test, Student's *t*‐test, and Kaplan–Meier assessed survival differences with log‐rank test for trend.

**Results:**

A total of 3406 patients were included with 1366 patients reported in the high‐risk group and 2040 patients in the low‐risk group. Quintile data demonstrated a stratification in survival based on socioeconomic status. Patients in more socially deprived counties were significantly (*p* = 0.035) more likely to have worse overall survival compared with those living in less deprived areas at 10‐year (87.3% vs 89.3%) follow‐up.

**Conclusions:**

10‐year overall and cancer‐specific survival data for patients with Wilms' tumor stratify by socioeconomic lines. This represents an area that needs to be addressed in this pediatric oncologic population. Patients from more socially deprived areas have significantly worse 10‐year overall survival rates and noticeably different 10‐year cancer‐specific survival rates.

## Graphical abstract

Wilm's tumor patients on a national level have significantly worse 10‐year overall survival rates and noticeably different 10‐year cancer‐specific survival rates. Disparities in healthcare outcomes among this pediatric oncologic population can be better addressed using social determination of health metrics.

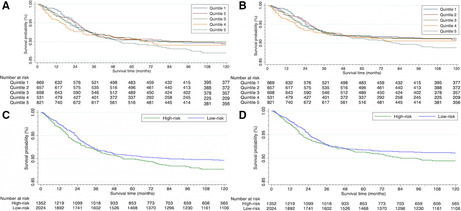

## 1 INTRODUCTION

Social determinants of health (SDoH), specifically inequities in terms of housing, food insecurity, health care access, and education is suggested to affect as much as 75% of children in the United States and represents a growing major public health concern.^1^ Despite a growing recognition of health care inequities in adults, the literature that evaluate SDoH and its effects on disease processes in the pediatric health is at a fledgling state. To this point, consensus does not exist for exactly which quantifiable SDoH measures potentially impact or could improve outcomes.^2^ The Social Deprivation Index (SDI) is a composite score based on demographic data commonly collected by the American Community Survey (ACS), commonly referred to as the Census. The SDI quantifies socioeconomic variation for income, education, employment, housing, household characteristics and transportation. The SDI is a score that based on a patient's geographic location, which can be applied in clinical practice to stratify disparities and health care needs and outcomes.^3^ Using deprivation‐based indexes from ACS data has been used before in oncological research. In a study that looked at the association of cancer screening practices based on area‐level social determinants, Kurani et al. found individuals living in areas of greater deprivation had lower cancer screening rates.^4^ Cheng et al. in 2021 found that neighborhood level deprivation led to worse oncological survival in several malignancies.^5^ No studies to date have applied similar SDoH stratification tools to measure oncological outcomes in the pediatric population.

Wilms' tumor (WT) represents the most common renal cancer in the pediatric population with a predicted five‐year overall survival of 90% with multimodal therapy.^6^ Although the prevalence of WT is only slightly elevated in Black patients, this population is disproportionally more likely to present with late‐stage disease according to the Therapeutically Applicable Research to Generate Effective Treatments registry.^7^ While race is the main studied disparity in WT patients, health care disparities are much more complex and multifaceted than one variable alone.^8^ The Surveillance of Epidemiology and End Results (SEER) database is one of the largest datasets describing cancer outcomes, representing roughly 30% of the United States population.^9^ Although the National Cancer Database is the largest clinical registry in the world, as the second largest dataset, SEER contains pertinent variables for a population level analysis using county level codes and survival outcomes.^10^ The purpose of this project is to stratify pediatric WT patient survival outcomes from available SEER data, by the SDI. The hypothesis is that there will be correlation between SDoH and survival, specifically that as the degree of social deprivation increases, there will be a negative impact demonstrated on survival outcomes.

## 2 METHODS

### 2.1 Data source and study population

The SEER 18 dataset is based on 18 population‐based cancer registries from 13 states, representing approximately 30% of the United States population.^9^ Since the data contained within SEER is deidentified, the retrospective cohort study was exempted for approval by the institutional review board at Nemours Children's Health (#1861345) prior to the study. After institutional review, SEER data was used to identify pediatric patients (<18 years old) from 1975 to 2016, who had a kidney topography code according to the International Classification of Diseases for Oncology (C64.9). The population was further defined using the ICD‐O‐3 histological code for WT (8960/8963) with a total of 3466 patients included in the initial study population. 54 patients with unavailable SDI data were removed from Alaska county (02900), Honolulu county (15912), Kauai county (15914), and Maui county (15915). Moreover, 6 patients with missing county data or survival data were excluded. This study followed the Strengthening the Reporting of Observational Studies in Epidemiology (STROBE) reporting guideline.

### 2.2 Social deprivation index

The study employed the latest available SDI scores (2015), which is based on county level data from the ACS, to measure the level of social deprivation experienced by each patient. The SDI measures 7 key demographic characteristics: percent living in poverty, percent with less than 12 years of education, percent single parent household, percent living in rented housing unit, percent living in overcrowded housing unit, percent of households without a car, and percent non‐employed adults under 65 years of age. The SDI is defined based on a range of 1 to 100 from national percentile rankings, with higher scores indicating worse social conditions. We defined SDI initially based on quintiles (Quintile 1 = 0–20, Quintile 2 = 21–40, Quintile 3 = 41–60, Quintile 4 = 61–80, and Quintile 5 = 81–100). After initial data review, SDI was further broken down based on the 60th percentile to define patients with high‐risk for worse outcomes in the studied population. In addition to the SDI variables defined by Butler et al., this study examined several other available variables to understand the degree of deprivation in a county.^3^ These included the percentage of the county population with high needs, defined as under the age of 5 or women between the ages of 15 and 44 years old.^3^ Additionally, the county data was evaluated for the percentage of the population who is foreign born or living linguistically isolated, defined by the ACS as living in a household where all members of the household aged 14 years or greater who speak a non‐English language and have less than fluent English.

### 2.3 Variables

The patient characteristics included age (measured in years), sex, race, ethnicity, tumor stage, and major treatment modality. The tumor stage in SEER is defined as localized: cancer confined to the primary site; regional: extension to nearby tissue or lymph nodes; or distant: spread to distant organs.^11^

### 2.4 Endpoints

The primary endpoint was overall survival, defined as the time from cancer diagnosis to death of any cause unless patient is lost to follow‐up. The secondary endpoint was WT cancer‐specific survival. Survival was evaluated at 10 years.

### 2.5 Statistical analysis

Patient demographic differences between the high‐risk group (>60 percentile) and low‐risk group (<60 percentile) were assessed using Fisher's exact test for categorical variables and Student's *t* test for continuous variables. The median and interquartile range (IQR) were used to record continuous variables while the frequency and percentage were used to record the categorical variables. The relation between social deprivation risk and patient demographics was compared using the chi‐square (χ^2^) test. A Kaplan–Meier analysis was performed to compare survival curves between the social deprivation groups. The log‐rank test was performed to compare *p*‐value survival curves, with log‐rank test for trend used for comparing quintile survival curves. To assess the effect of SDI factors on overall survival, a multivariate Cox proportional hazards regression model was performed using patient characteristics. For all tests, significance was defined based on a two‐tail *p* < 0.05. Analysis was conducted using the Statistical Package for the Social Sciences version 28.0 (IBM Corporation) and Stata version 17 (Stata Corporation). The R version 4.1.0 was utilized with the RCommander package and EZR PlugIn.^12^

## 3 RESULTS

3406 patients met inclusion criteria. A summary of the patient demographics can be found in Table 1. In the entire cohort, WT patients had a median age of 3 years old (IQR: 1–5) with 1845 female patients (54.2%). Most patients were White representing 2635 patients (77.2%) followed by Black representing 582 patients (17.1%). 36 patients in the study population had an unknown race. 754 patients (22.1%) identified as Hispanic ethnicity. Majority of WT patients received surgery, with 89 patients (2.6%) not undergoing surgery. The overall 10‐year survival was 88.5% in the entire cohort with a 10‐year cancer‐specific survival rate of 91.1%.

**TABLE 1:** Baseline patient demographics in patients with a Wilms' tumor diagnosis

*n* = 3406	Quintile 1 (674)	Quintile 2 (662)	Quintile 3 (704)	Quintile 4 (539)	Quintile 5 (827)	Total (%)	*p* value
Age (in Years)	3 [1–5]	3 [1–5]	3 [1–5]	3 [1–5]	3 [1–5]	3 [1–5]	0.57
Sex (Female)	363 (53.9%)	349 (52.7%)	401 (57.0%)	263 (48.8%)	469 (56.7%)	1845 (54.1%)	0.024
Race							
White	607 (90.1%)	546 (82.5%)	550 (78.1%)	381 (70.7%)	551 (66.6%)	2635 (77.4%)	<0.001
Black	43 (6.4%)	71 (10.7%)	105 (14.9%)	137 (25.4%)	226 (27.3%)	582 (17.0%)	
Other	18 (2.7%)	38 (5.7%)	41 (5.8%)	17 (3.2%)	43 (5.2%)	157 (4.6%)	
Ethnicity							
Hispanic	50 (7.4%)	92 (13.9%)	168 (23.9%)	115 (21.3%)	329 (39.8%)	754 (22.1%)	<0.001
Tumor stage							
Localized	296 (43.9%)	277 (41.8%)	289 (41.1%)	223 (41.4%)	343 (41.5%)	1428 (41.9%)	0.725
Regional	188 (27.9%)	209 (31.6%)	204 (29.0%)	145 (26.9%)	228 (27.6%)	974 (28.6%)	
Distant	149 (22.1%)	139 (21.0%)	159 (22.6%)	132 (24.5%)	195 (23.6%)	774 (22.7%)	
Unstaged	41 (6.1%)	37 (5.6%)	52 (7.4%)	39 (7.2%)	61 (7.4%)	230 (6.8%)	
Treatment							
Surgery	664 (98.5%)	646 (97.6%)	689 (97.9%)	517 (95.9%)	801 (96.9%)	3317 (97.4%)	0.048
No Surgery	10 (1.5%)	16 (2.4%)	15 (2.1%)	22 (4.1%)	26 (3.1%)	89 (2.6%)	

The SEER WT cohort was broken down by the factors that make up the SDI. This puts 13.9% (4.4–37.5%) of the population living in an area defined as 100% below the Federal Poverty Level. 18.3% (6.9–30.5%) of the population dwell in an area from a single‐parent household. The median high school dropout rate is 12.6% (1.6–34.9%). 12.6% (0.6–32.5%) of WT patients live in an area without a car in the household. The median number of households renting, as opposed to owning the home they live in, is 37.0% (14.2–68.7%) in our population. Moreover, the median number of households living in a crowded area is 3.0% (0.2–12.7%) in the cohort. The median percentage of non‐employed individuals by county, who are capable of working, but for any reason are unable to is 8.8% (1.6–21.3%) in the studied population. The median percentage of high needs individuals is 40.0% (26.8–49.2%) and the median percentage of foreign‐born individuals by county is 15.1% (0–41.9%). The median percentage of linguistically isolated households in the county is 4.9% (0–21.3%) in this studied population.

The patient population were initially separated based on SDI score by quintiles. Across quintiles, WT patients were on average 3 years in age (IQR: 1–5). Patients in the most socially deprived areas were significantly (*p* = 0.024) more likely to identify as female (25.4% vs 19.7%). Compared to patient in the least socially deprived areas, patients living in the most deprived counties (Quintile 5) were significantly (*p* < 0.001) more likely to be Black (38.8% vs 7.4%) or Other (27.4% vs 11.5%). Compared to patients living in the least deprived counties, patients living in the most deprived counties were significantly (*p* < 0.001) more likely to be of Hispanic ethnicity (43.6% vs 6.6%). In the most deprived counties, patients were significantly (*p* = 0.048) more likely to not receive surgery as primary treatment (3.1% vs 1.5%). No significant differences were found based on tumor stage in SEER at the time of initial presentation.

**FIGURE 1:**
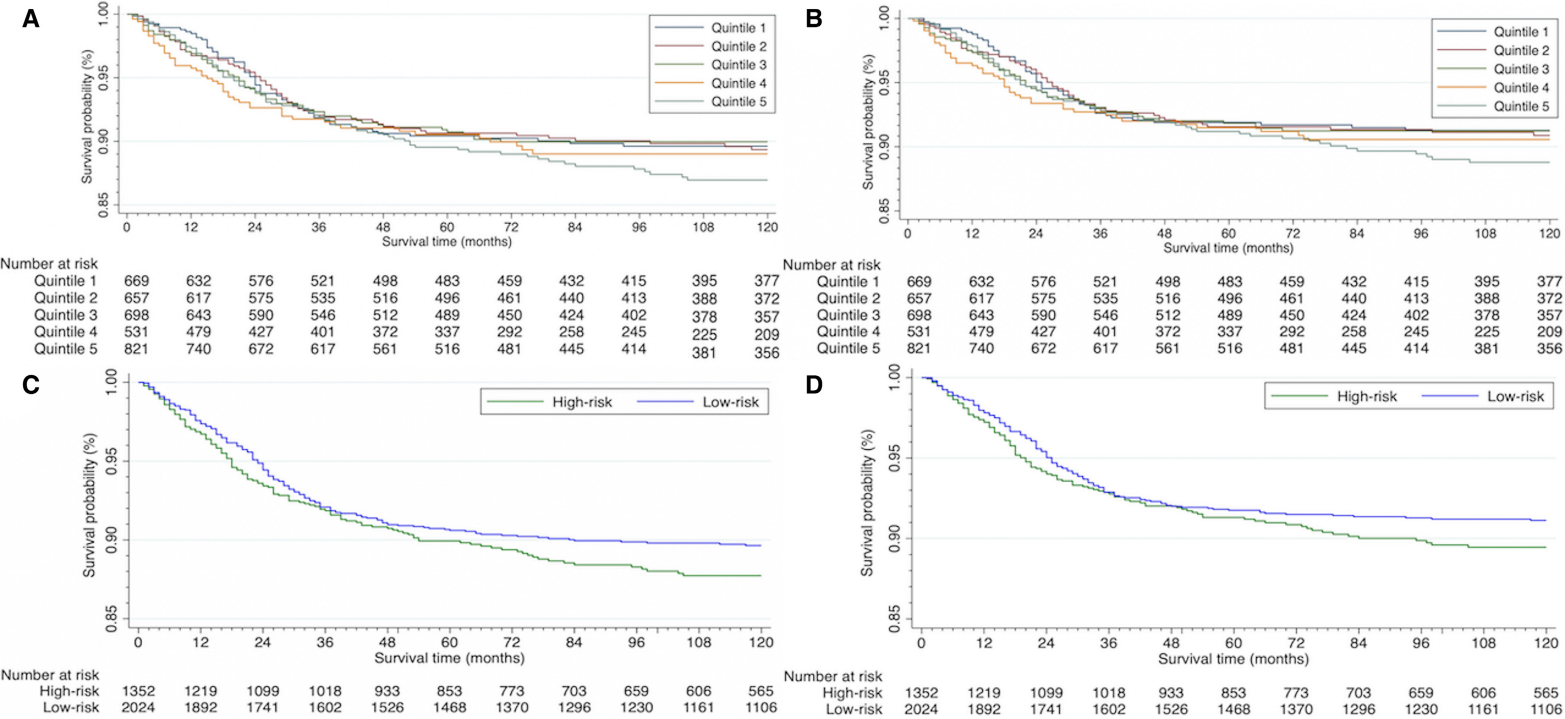
SDI quintiles for overall survival (A) and cancer‐specific survival (B). SDI risk (defined based on the 60th percentile) for overall survival (C) and cancer‐specific survival (D).

Evaluating the survival curves in Figure 1, as the curves reach the 10‐year mark, visually apparent differences become present between the quintiles. The 3 least deprived quintiles cluster and the 2 more deprived quintiles begin to cluster. The survival curves also begin to separate. Table 2 demonstrates that as the quintiles become increasingly more deprived compared overall survival for Q1, the *p*‐value steadily improves, decreasing from *p* = 0.97 (Q2), *p* = 0.72 (Q3), *p* = 0.35 (Q4) to *p* = 0.14 (Q5). Looking at the data in Table 3, overall survival rates between Q1 (least deprived) and Q5 (most deprived) are 89.2% (95% CI: 84.4–91.5%) versus 86.6% (95% CI: 83.8%–89.0%) respectively. A similar difference is seen in cancer‐specific survival rates. The quintile data does not reach a level of statistical significance despite the separation in survival curves.

**TABLE 2:** Adjusted hazard ratio between social deprivation index (SDI)

Overall survival	Hazard ratio	*p* value	Cancer‐specific survival	Hazard ratio	*p* value
**SDI quintile**			**SDI quintile**		
Quintile 1	1 [Reference]	NA	Quintile 1	1 [Reference]	NA
Quintile 2	1.00 [0.73–1.38]	0.97	Quintile 2	1.03 [0.71–1.47]	0.89
Quintile 3	0.94 [0.68–1.30]	0.72	Quintile 3	0.99 [0.69–1.42]	0.95
Quintile 4	1.18 [0.84–1.65]	0.35	Quintile 4	1.22 [0.84–1.78]	0.3
Quintile 5	1.25 [0.93–1.69]	0.14	Quintile 5	1.27 [0.91–1.77]	0.17
**At‐risk SDI**			**At‐risk SDI**		
Low‐risk	1 [Reference]	NA	Low‐risk	1 [Reference]	NA
High‐risk	1.25 [1.02–1.53]	0.035	High‐risk	1.22 [0.99–1.56]	0.058

**TABLE 3:** 10‐Year overall and cancer‐specific survival by social deprivation index (SDI)

Overall survival	10 year % (95% CI)	*p* value	Cancer‐specific survival	10 Year % (95% CI)	*p* value
**SDI quintile**		0.31	**SDI quintile**		0.45
Quintile 1	89.2% (86.4–91.5%)		Quintile 1	91.0% (88.4–93.0%)	
Quintile 2	88.9% (86.1–91.2%)		Quintile 2	90.5% (87.8–92.6%)	
Quintile 3	89.7% (87.0–91.8%)		Quintile 3	91.1% (88.6–93.1%)	
Quintile 4	88.4% (85.0–91.0%)		Quintile 4	89.9% (86.8–92.3%)	
Quintile 5	86.6% (83.8–89.0%)		Quintile 5	88.5% (85.8–90.7%)	
**Risk**		0.035	**Risk**		0.058
Low‐risk	89.3% (87.8–90.6%)		Low‐risk	90.9% (89.4–92.1%)	
High‐risk	87.3% (85.2–89.1%)		High‐risk	89.0% (87.0–90.7%)	

The above noted visual differences in quintile survival curves and the changes in *p*‐values, led to dichotomizing the patient population into two subcategories, a high‐risk (>60 percentile/more deprived) population and low‐risk (<60 percentile/less deprived) cohorts based on SDI scores. A total of 1366 patients (40.1%) were reported in the high‐risk group and 2040 patients (59.9%) in the low‐risk social deprivation group. Patients in the high‐risk population and low‐risk population showed comparable numbers by gender, age, and tumor stage. Compared to the low‐risk population, patients in the high‐risk population were significantly (*p* < 0.001) more likely to be reported as Black (26.6% vs 10.7%). Compared to the low‐risk population, patients in the high‐risk population were significantly (*p* < 0.001) more likely to be classified as Hispanic ethnicity (32.5% vs 15.2%). The high‐risk group was significantly (*p* = 0.008) more likely to not receive surgery (3.5% vs 2.0%).

Patients in the high‐risk group were 1.25 times (HR: 1.25 [95% CI: 1.02–1.35]) significantly (*p* = 0.035) more likely to experience mortality compared to the low‐risk group (Table 2). At 10‐year follow‐up, patients in the high‐risk population had an overall survival of 87.3% (95% CI: 85.2–89.1%) compared to 89.3% (95% CI: 87.8–90.6%) in the low‐risk population (Table 3). Cancer‐specific survival rates between the two groups were noticeably different but statistically non‐significant (*p* = 0.058). At 10‐year follow‐up, patients the high‐risk population had a cancer‐specific survival of 89.0% (95% CI: 87.0–90.7%) compared to 90.9% (95% CI: 89.4–92.1%) in the low‐risk population.

## 4 DISCUSSION

Considering that a high percentage of health related outcomes are affected by SDoH, medical care alone is insufficient to address successful health outcomes for all.^13^ Despite evidence recognizing the impact of SDoH, there are currently no universal screening tools used by clinicians in the pediatric population.^14^ However, institutional based studies have reported that universal screening of SDOH is feasible in the United States health care system, especially in a value‐based model.^15^, ^16^ On a global level, WT patients from low‐income countries have a low 3‐year overall survival rate of 50% due mostly to limited access to care and lack of facilities for treatment.^17^ Although survival has improved in pediatric oncology patients in the United States, oncological survival disparities are still a major public health issue. Patients of lower socioeconomic status or from a disenfranchise racial/ethnic group having lower rates of overall survival and higher rate of relapse across pediatric cancers.^18^

The average pediatric patient with a WT diagnosis has a real risk of experiencing social barriers that may impact care. In the SEER WT population, on average 13.9% of patients live in an area defined as 100% below the Federal Poverty Level. A pediatric cancer diagnosis is a significant socioeconomic burden on the family, with advanced disease known to represent the largest burden.^19^ In a multicenter study from 2000–2010, children living in poverty with a leukemia diagnosis were at a disproportionately higher risk of experiencing relapse compared to low‐poverty areas.^20^ In our population, we found the impact of poverty manifested in several significant ways on a county level including the high school dropout rate (12.6%), lack of car in the household (12.6%), household members being non‐employed (8.8%), linguistic isolated (4.9%) or lack of social community due to being foreign born (15.1%). These social conditions represent tangible barriers to medical care including lack of health care resource access, lack of medical knowledge, mistrust between patient and the medical system, and being unable to travel or follow‐up on care.^21^ Social conditions related to health and nutrition may similarly have an impact on epigenetics, portending higher disease risk.^22^, ^23^

Patients with a WT diagnosis defined in the high‐risk population are significantly more likely to be racial or ethnic minorities in the most socially deprived communities. In this study population, patients in the high‐risk cohort were 2 times more likely to be Black (26.6% vs 10.7%) or of Hispanic ethnicity (32.5% vs 15.2%) compared to low‐risk patients. Patients of Hispanic ethnicity have significantly lower rates of cancer‐specific survival compared to non‐Hispanic Caucasian counterparts in national studies.^24^ While there are no national studies that report on WT survival differences based on racial disparities, one major academic institutional study found that Black patients with a WT diagnosis had noticeably lower, yet non‐significant, differences in overall survival compared to White patients.^25^ Disparities in outcomes for WT patients who are racial and ethnic minorities is deeply rooted in social and economic conditions, which represent an opportunity for better addressment by clinicians.

The SDI is a sensitive quantitative metric that can potentially be used by clinicians to account for social disparities in the pediatric population based only on the county where the patient resides. Patients with a WT diagnosis from the most socially deprived counties, defined in this population as above the 60th percentile, had significantly (*p* = 0.035) lower 10‐year overall survival rates (89.3% [95% CI: 87.8–90.6%] vs 87.3% [95% CI: 85.2–89.1]). These patients also appear to have worse cancer‐specific survival (90.9% [95% CI: 89.4–92.1%] vs 89.0% [95% CI: 87.0–90.7%]); this did not reach the level of statistical significance (*p* = 0.058). This data demonstrates that patients from more socially deprived counties consistently showed lower overall survival and cancer‐specific survival outcomes. In other countries such as the United Kingdom and New Zealand, population‐level indices based around social determinants of health are used to direct interventions, as part of risk assessment tools, and as part of insurance reimbursement.^26^ Population‐level indices can be used in the United States in a similar capacity to improve healthcare outcomes especially in the pediatric oncology population.

The demonstrated differences in survival when the WT cohort was subdivided into quintile data were not found to be statistically significant despite the obvious visual differences between the groups. The lack of statistical significance is likely accounted for by two major factors. Firstly, WT is rare and the sample size is small for detecting small differences. Secondly, the survival rates are relatively high amongst the cohort. These cause tight groupings to appear in survival data, but the visual trend and pattern in how the hazard ratios, p‐values, and statistics shifted when the cohort became less deprived is valuable data to present. Slight variations in pediatric populations with high survival rates require a large sample size to detect differences. As reported in a systematic review on health inequalities amongst pediatric oncology patients from high‐income countries by Mogensen et al., studies on pediatric cancer with small sample sizes or with a very high survival rate created a lack of statistical power; however, the overlying theme remains the same: in the majority of studies, patients with worse socioeconomic predictors have worse survival outcomes.^27^ Amongst the demographic characteristics we studied in our population, we found patients with a worse social condition whether that be based on poverty, low educational status below high school, being non‐employed, dwelling in a crowded housing or renter occupied area, being a single‐parent households, or being without a car for transportation demonstrated lower survival rates across the board comparing the least deprived to most deprived subgroups.

This study has several limitations. SDI is one composite score of social deprivation based on several key demographic factors, but it is not a comprehensive measure of social disparity at a patient experience level. While it is used as an accepted index in other published studies, drilling down to even the block level to measure social disparities would be more specific and ideal.^28^, ^29^ Further studies would be needed to better evaluate the application of all these indices conclusively and apply them to other oncological conditions and health issues. Additionally, outcomes were based on county data, which may not be representative of individual socioeconomic status especially as in any county there are varying degrees of affluence and access across different neighborhoods. As this study was based in pediatric patients with a WT diagnosis this may not be generalizable to all pediatric cancers and so caution should be taken in extrapolating from this data from a SDoH perspective. While the SEER database has a large patient sample, this study was limited to only the patients that were recorded in the registry and as such may not represent the entire pediatric population. The SEER population does not provide data on whether patients were enrolled in Children's Oncology Group (COG) protocols and completed therapy. Faulk et al. found racial/ethnic groups and county‐level socioeconomic factors were similar between pediatric oncology patients in SEER and patients enrolled in the COG therapeutic trials.^30^

In summary, pediatric patients with a WT diagnosis experience SDoH disparities that affect health care outcomes. The SDI was shown to be a useful tool to stratify potential SDoH variability within patient populations and may be useful as a tool in value‐based healthcare models to work to improve healthcare outcomes. Patients from the most socially deprived areas with WT had significantly (*p* = 0.035) worse 10‐year overall survival rates. There was also a noticeable difference in 10‐year cancer‐specific survival rates between patients more potentially exposed to negative SDoH variable in this studied population. While survival differences are not huge, they were by no means trivial to pediatric patients and their families. More comprehensive health care policies and practices focusing on reducing SDoH disparities such as seen in the WT population should be implemented. Medical care providers need to focus on pediatric patients who are most vulnerable, specifically coming from more socially deprived geographic areas and backgrounds.

## AUTHOR CONTRIBUTION

Victor Chalfant: Data curation, formal analysis, software, and writing – original draft, and writing – review and editing. Carlos Riveros: Methodology, formal analysis, and writing – review and editing. Andrew A. Stec: Conceptualization, data curation, methodology, project administration, writing – original draft, and writing – review and editing.

## Funding information

There was no study sponsor or extramural funding.

## CONFLICT OF INTEREST

The authors declare no conflicts of interest.

## ETHICS APPROVAL

Ethics approval was obtained from Nemours Children's Health (IRB#1861345).

## PRECIS

Survival disparities exist amongst Wilms' Tumor patients based on factors related to social determinants of health. More comprehensive health care policies and practices are needed in this pediatric oncologic population to address these differences in survival.

## Data Availability

The data that support the findings of this study are available from the corresponding author upon reasonable request.
